# Supplementary material for: Acknowledging the role of patient heterogeneity in hospital outcome reporting: Mortality after acute myocardial infarction in five European countries
Source: PLoS One. 2020 Feb 6;15(2):e0228425. doi: 10.1371/journal.pone.0228425 (PMC7004308; doi:10.1371/journal.pone.0228425)
Supplement: S1 Appendix — Inclusion and exclusion criteria and codes for episode selection. (DOCX) [file pone.0228425.s001.docx]

**S1. Description of selected codes**

Table 1. Inclusion and exclusion criteria and codes for episode selection.

|  | Primary diagnostic | | Secondary diagnostic | |
| --- | --- | --- | --- | --- |
|  | Inclusion codes | Exclusion codes[s1] | Inclusion codes | Exclusion codes |
| Denmark (ICD10) | I22* I23* | O00* O99* | --- | O00* O99* |
| Portugal (ICD9-MC) | 410 | 630-677 | --- | 630-677 |
| Slovenia (ICD10) | I22* I23* | O00* O99* | --- | O00* O99* |
| Spain (ICD9-MC) | 410 | 630-677 | --- | 630-677 |
| Sweden (ICD10) | I22* I23* | O00* O99* | --- | O00* O99* |

Inclusion codes correspond to Acute Myocardial infarction diagnosis

ICD-10 cod. I22*: Subsequent ST elevation (STEMI) and non-ST elevation (NSTEMI) myocardial infarction

ICD-10 cod. I23*: Certain current complications following ST elevation (STEMI) and non-ST elevation (NSTEMI) myocardial infarction (within the 28-day period)

ICD-9 cod. 410: Acute myocardial infarction

Exclusion codes correspond to MDC14: Pregnancy, Childbirth and the puerperium.

ICD-10 cod. O00*: Ectopic pregnancy

ICD-10 cod. O99*: Other maternal diseases classifiable elsewhere but complicating pregnancy, childbirth and the puerperium

ICD-9 cod. from 630 to 677: (630-639 Ectopic and Molar Pregnancy, and Other Pregnancy With Abortive Outcome; 640-649 Complications Mainly Related To Pregnancy; 650-659 Normal Delivery, And Other Indications For Care In Pregnancy, Labour, And Delivery; 660-669 Complications Occurring Mainly In The Course Of Labour And Delivery; 670-677 Complications Of The Puerperium).
