# Supplementary material for: Acknowledging the role of patient heterogeneity in hospital outcome reporting: Mortality after acute myocardial infarction in five European countries
Source: PLoS One. 2020 Feb 6;15(2):e0228425. doi: 10.1371/journal.pone.0228425 (PMC7004308; doi:10.1371/journal.pone.0228425)
Supplement: S2 Appendix — (DOCX) [file pone.0228425.s002.docx]

**S2. Survival curves testing differential underreporting in CHF patients**

Fig 1. Survival curve - Denmark


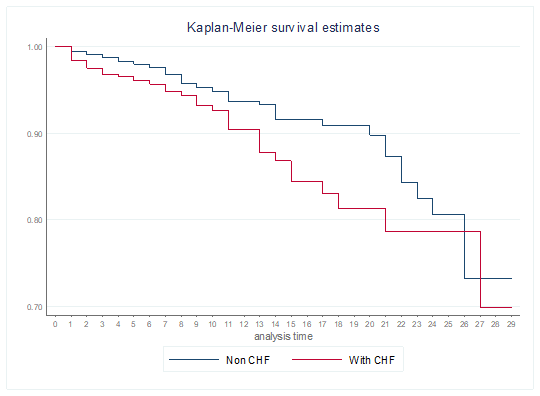


Table 1. Denmark - survivor and cumulative hazard functions after Acute Myocardial Infarction for the two categories, with and without CHF, using the specified comparison times

|  | Survivor function | | Nelson-Aalen cum. Hazard | |
| --- | --- | --- | --- | --- |
| time | wo CHF | w CHF | wo CHF | w CHF |
| 1 | 0.994 | 0.9843 | 0.006 | 0.0157 |
| 4 | 0.9834 | 0.9659 | 0.0167 | 0.0345 |
| 7 | 0.9682 | 0.9484 | 0.0323 | 0.0527 |
| 10 | 0.9486 | 0.9258 | 0.0526 | 0.0767 |
| 13 | 0.9331 | 0.8784 | 0.069 | 0.1286 |
| 16 | 0.9159 | 0.8449 | 0.0874 | 0.167 |
| 19 | 0.9093 | 0.8141 | 0.0946 | 0.2038 |
| 22 | 0.8463 | 0.7887 | 0.1654 | 0.2351 |
| 25 | 0.811 | 0.7887 | 0.2076 | 0.2351 |
| 28 | 0.7462 | 0.7098 | 0.2876 | 0.3351 |
| 31 | . | . | . | . |

Fig 2. Survival Curve - Portugal


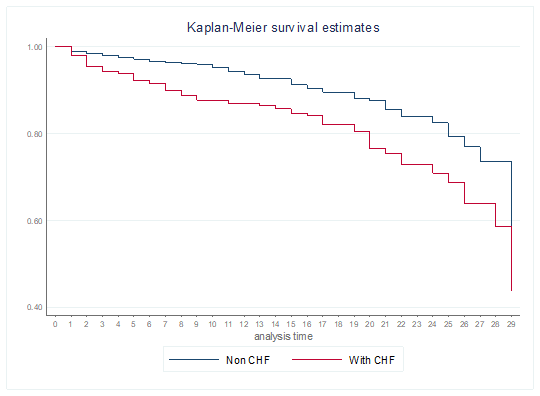


Table 2. Portugal - survivor and cumulative hazard functions after Acute Myocardial Infarction for the two categories, with and without CHF, using the specified comparison times

|  | Survivor function | | Nelson-Aalen cum. Hazard | |
| --- | --- | --- | --- | --- |
| time | wo CHF | w CHF | wo CHF | w CHF |
| 1 | 0.9895 | 0.979 | 0.0105 | 0.021 |
| 4 | 0.9751 | 0.9382 | 0.0252 | 0.0632 |
| 7 | 0.9639 | 0.8995 | 0.0366 | 0.105 |
| 10 | 0.9524 | 0.8782 | 0.0486 | 0.1288 |
| 13 | 0.9258 | 0.8657 | 0.0768 | 0.1431 |
| 16 | 0.9034 | 0.8423 | 0.1012 | 0.1703 |
| 19 | 0.8818 | 0.8079 | 0.1252 | 0.2117 |
| 22 | 0.8408 | 0.7357 | 0.1724 | 0.3035 |
| 25 | 0.7988 | 0.7002 | 0.2228 | 0.3524 |
| 28 | 0.7495 | 0.6191 | 0.2855 | 0.4718 |
| 31 | . | . | . | . |

Figure 3. Survival Curve - Slovenia


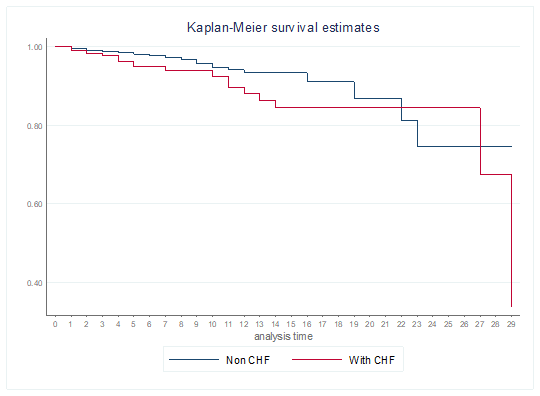


Table 3. Slovenia - survivor and cumulative hazard functions after Acute Myocardial Infarction for the two categories, with and without CHF, using the specified comparison times

|  | Survivor function | | Nelson-Aalen cum. Hazard | |
| --- | --- | --- | --- | --- |
| time | wo CHF | w CHF | wo CHF | w CHF |
| 1 | 0.9943 | 0.989 | 0.0057 | 0.011 |
| 4 | 0.9846 | 0.963 | 0.0155 | 0.0375 |
| 7 | 0.9721 | 0.9388 | 0.0282 | 0.0628 |
| 10 | 0.9465 | 0.9254 | 0.0548 | 0.0771 |
| 13 | 0.9341 | 0.8651 | 0.068 | 0.1437 |
| 16 | 0.9104 | 0.8454 | 0.0933 | 0.1664 |
| 19 | 0.8671 | 0.8454 | 0.1409 | 0.1664 |
| 22 | 0.8129 | 0.8454 | 0.2034 | 0.1664 |
| 25 | 0.7451 | 0.8454 | 0.2868 | 0.1664 |
| 28 | 0.7451 | 0.7045 | 0.2868 | 0.3331 |
| 31 | . | . | . | . |

Fig 4. Survival Curve - Spain


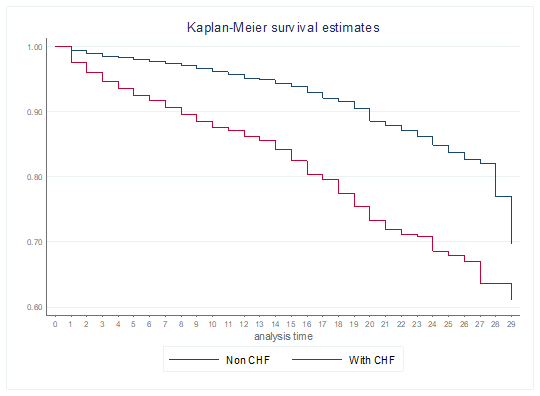


Table 4. Spain - survivor and cumulative hazard functions after Acute Myocardial Infarction for the two categories, with and without CHF, using the specified comparison times

|  | Survivor function | | Nelson-Aalen cum. Hazard | |
| --- | --- | --- | --- | --- |
| time | wo CHF | w CHF | wo CHF | w CHF |
| 1 | 0.9932 | 0.9763 | 0.0068 | 0.0237 |
| 4 | 0.9825 | 0.9358 | 0.0176 | 0.0658 |
| 7 | 0.9742 | 0.9071 | 0.0261 | 0.0968 |
| 10 | 0.9614 | 0.8774 | 0.0393 | 0.1299 |
| 13 | 0.9489 | 0.8574 | 0.0523 | 0.1529 |
| 16 | 0.9304 | 0.8065 | 0.072 | 0.2134 |
| 19 | 0.9059 | 0.7604 | 0.0985 | 0.2716 |
| 22 | 0.8753 | 0.7207 | 0.1327 | 0.3247 |
| 25 | 0.8442 | 0.6915 | 0.1686 | 0.3655 |
| 28 | 0.7936 | 0.6587 | 0.2293 | 0.4134 |
| 31 | . | . | . | . |

Figure 5. Survival Curve - Sweden


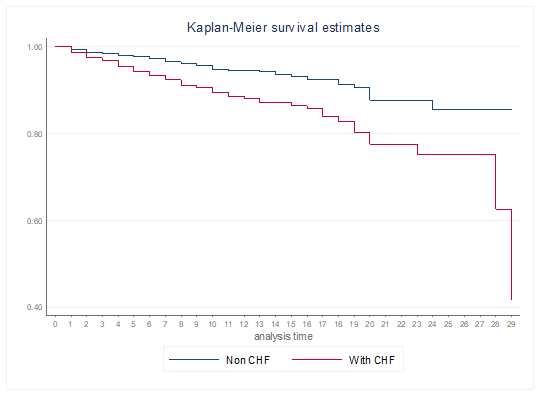


Table 5. Sweden - survivor and cumulative hazard functions after Acute Myocardial Infarction for the two categories, with and without CHF, using the specified comparison times

|  | Survivor function | | Nelson-Aalen cum. Hazard | |
| --- | --- | --- | --- | --- |
| time | wo CHF | w CHF | wo CHF | w CHF |
| 1 | 0.992 | 0.9866 | 0.008 | 0.0134 |
| 4 | 0.9802 | 0.9544 | 0.0199 | 0.0464 |
| 7 | 0.9648 | 0.9233 | 0.0357 | 0.0793 |
| 10 | 0.9484 | 0.8962 | 0.0529 | 0.109 |
| 13 | 0.9432 | 0.8717 | 0.0584 | 0.1365 |
| 16 | 0.9238 | 0.8578 | 0.079 | 0.1525 |
| 19 | 0.9061 | 0.8065 | 0.0983 | 0.2135 |
| 22 | 0.8786 | 0.7809 | 0.1286 | 0.2452 |
| 25 | 0.8577 | 0.7592 | 0.1524 | 0.273 |
| 28 | 0.8577 | 0.6833 | 0.1524 | 0.373 |
| 31 | . | . | . | . |
