# Supplementary material for: Acknowledging the role of patient heterogeneity in hospital outcome reporting: Mortality after acute myocardial infarction in five European countries
Source: PLoS One. 2020 Feb 6;15(2):e0228425. doi: 10.1371/journal.pone.0228425 (PMC7004308; doi:10.1371/journal.pone.0228425)
Supplement: Attachment — Submitted filename: PONE-D-19-28081R1_Response-to-reviewers_20200110.docx [file pone.0228425.s003.docx]

**Ref: PONE-D-19-28081-R1**

**Responses to the first round of reviews**

**Version 2020 01 10**

Dear editor,

Thank you very much for allowing us to submit a reviewed version of our manuscript. Please, find below our replies to your concerns and the first reviewer’s comments in a case-by-case manner. Those points that implied a change in the manuscript have been conveniently highlighted.

**Editor’s concerns and journal’s requirements:**

**2**. We note that you have indicated that data from this study are available upon request. PLOS only allows data to be available upon request if there are legal or ethical restrictions on sharing data publicly. In your revised cover letter, please address the following prompts:

a) If there are ethical or legal restrictions on sharing a de-identified data set, please explain them in detail (e.g., data contain potentially identifying or sensitive patient information) and who has imposed them (e.g., an ethics committee). Please also provide contact information for a data access committee, ethics committee, or other institutional body to which data requests may be sent (We will update your Data Availability statement on your behalf to reflect the information you provide).

**Response:** As required, we have added an informative paragraph in the revised cover letter as follows:

*“The access to pseudoanonimised patient-level administrative data (patient episodes: hospital admissions and discharges) supporting the manuscript findings is restricted to avoid data misuse in accordance with the System Level Security Policy of the Unit for Health Services and Policy Research at the Institute for Health Sciences in Aragon (IACS) (Available at: www.atlasvpm.org/grupo-coordinador/ARiHSPinformationsecuritypolicy) within the framework of the Spanish legal system, in particular, Regulation (EU) 2016/679, the Law 3/2018 on Personal Data Protection, the Law 14/2007 on Biomedical Research and the Law 37/2007 and Law 18/2015 on the Public Sector Information Reuse. Consequently, we have specified that only aggregated data may be accessed, upon request, throughout our data sharing agreement, by contacting the senior author of the manuscript Enrique Bernal-Delgado (ebernal.iacs@aragon.es) and/or the legal officer Ramón Launa-Garcés (*[*rlaunag.iacs@aragon.es*](mailto:rlaunag.iacs@aragon.es)*)”*

**5**. Thank you for stating in your Funding Statement:

"*The ECHO Project received funding from the 7th Framework Programme of the European Union (2010-2014) with Grant HEALTH-F3-2010-242189 and from the European Union’s Health Programme (2014-2020) with Grant 664691/BRIDGE Health. On the other hand, the authors Enrique Bernal-Delgado, Francisco Estupiñán-Romero and Micaela Comendeiro-Maaløe have been partially funded by the Institute for Health Carlos III through a public competitive grant (RD16/0001/0007) as part of the network for Health Services Research on Chronic Patients (REDISSEC). The funders had no role in study design, data collection and analysis, decision to publish, or preparation of the manuscript*."

i) Please provide an amended statement that declares *all* the funding or sources of support (whether external or internal to your organization) received during this study, as detailed online in our guide for authors at http://journals.plos.org/plosone/s/submit-now. Please also include the statement “There was no additional external funding received for this study.” in your updated Funding Statement.

ii) Please include your amended Funding Statement within your cover letter. We will change the online submission form on your behalf.

**Response:** As required, we have added an amended statement in the revised cover letter as follows:

“*The ECHO Project received funding from the 7th Framework Programme of the European Union (2010-2014) with Grant HEALTH-F3-2010-242189 and from the European Union’s Health Programme (2014-2020) with Grant 664691/BRIDGE Health. Additionally, the authors Enrique Bernal-Delgado, Francisco Estupiñán-Romero and Micaela Comendeiro-Maaløe have been partially funded by the Institute for Health Carlos III through a public competitive grant (RD16/0001/0007) as part of the network for Health Services Research on Chronic Patients (REDISSEC). The funding sources received during this study played no role in the study design, data collection or the analysis, decision to publish, or preparation of the manuscript. There was no additional external funding received for this study*”

6. Thank you for stating the following in the Acknowledgments Section of your manuscript:

"The ECHO Project received funding from the 7th Framework Programme of the European Union (2010-2014) with grant HEALTH-F3-2010-242189 and from the European Union’s Health Programme (2014-2020) with grant 664691/BRIDGE Health."

We note that you have provided funding information that is not currently declared in your Funding Statement. However, funding information should not appear in the Acknowledgments section or other areas of your manuscript. We will only publish funding information present in the Funding Statement section of the online submission form.

* Please remove any funding-related text from the manuscript and let us know how you would like to update your Funding Statement. Currently, your Funding Statement reads as follows:

"*The ECHO Project received funding from the 7th Framework Programme of the European Union (2010-2014) with Grant HEALTH-F3-2010-242189 and from the European Union’s Health Programme (2014-2020) with Grant 664691/BRIDGE Health. On the other hand, the authors Enrique Bernal-Delgado, Francisco Estupiñán-Romero and Micaela Comendeiro-Maaløe have been partially funded by the Institute for Health Carlos III through a public competitive grant (RD16/0001/0007) as part of the network for Health Services Research on Chronic Patients (REDISSEC). The funders had no role in study design, data collection and analysis, decision to publish, or preparation of the manuscript*".

**Response:** all the funding information has been removed from the manuscript as required. The funding information has also been updated and included in the revised cover letter as required in point 5.

**Reviewer’s concerns:**

Reviewer #1

The introduction is difficult to follow and needs to be simplified for the average reader. For instance, Studies assessing the effect of Post MI heterogeneities have largely focused on mortality as the primary outcome. Differences in post MI survival can be explained by disparities in available resources and medical care.

**Response:** Thank you for your suggestion, although we are afraid that by using other terms as you propose in your example, the meaning of the conceptualisation of what we methodologically develop later in the paper could be changed. Nevertheless, we believe that the corrections made in the introduction once the manuscript has been proofread by a native speaker, may ease comprehension.

The manuscript is fraught with some minor grammatical errors and needs to be thoroughly proofread before submission.

**Response:** thank you for the suggestion. As mentioned before, the manuscript has been proofread and corrected by a native speaker.

Flowchart of patient participation, exclusion and inclusion should be included in the manuscript if possible

**Response:** Thank you for the suggestion. A flowchart has been included at the beginning of the methods section, now named as figure 1.
